# PeaKDEck: a kernel density estimator-based peak calling program for DNaseI-seq data

**DOI:** 10.1093/bioinformatics/btt774

**Published:** 2014-01-08

**Authors:** Michael T. McCarthy, Christopher A. O’Callaghan

**Affiliations:** Wellcome Trust Centre for Human Genetics, Nuffield Department of Medicine, University of Oxford, Roosevelt Drive, Oxford, OX3 7BN, UK

## Abstract

**Summary:** Hypersensitivity to DNaseI digestion is a hallmark of open chromatin, and DNaseI-seq allows the genome-wide identification of regions of open chromatin. Interpreting these data is challenging, largely because of inherent variation in signal-to-noise ratio between datasets. We have developed PeaKDEck, a peak calling program that distinguishes signal from noise by randomly sampling read densities and using kernel density estimation to generate a dataset-specific probability distribution of random background signal. PeaKDEck uses this probability distribution to select an appropriate read density threshold for peak calling in each dataset. We benchmark PeaKDEck using published ENCODE DNaseI-seq data and other peak calling programs, and demonstrate superior performance in low signal-to-noise ratio datasets.

**Availability and implementation:** PeaKDEck is written in standard Perl and runs on any platform with Perl installed. PeaKDEck is also available as a standalone application written in Perl/Tk, which does not require Perl to be installed. Files, including a user guide, can be downloaded at: www.ccmp.ox.ac.uk/peakdeck.

**Contact:** chris.ocallaghan@ndm.ox.ac.uk

**Supplementary information:** Supplementary data are available at *Bioinformatics* online.

## 1 INTRODUCTION

DNaseI hypersensitivity analysis can be used to map sites of open chromatin in genomic DNA (Wu, 1980). Hypersensitivity of DNA to digestion by DNaseI arises when nucleosomal histone proteins are displaced from chromatin, leaving a region of ‘naked’ nucleosome-free DNA that is accessible to the DNaseI enzyme (Owen-Hughes and Workman, 1996). Histone displacement and consequent DNaseI hypersensitivity characteristically occur at promoter and enhancer sites (Song *et al.*, 2011), allowing the sequence-specific binding of proteins, such as transcription factors to the DNA. Recently, advances in high-throughput sequencing methods have been applied to DNaseI hypersensitivity testing [DNaseI-seq; Supplementary Information, Section S1; (Boyle *et al.*, 2008a; Hesselberth *et al.*, 2009)]). With DNaseI-seq, regulatory DNA fragments at accessible open chromatin sites are released by ‘two-hit’ digestion. These fragments are sequenced using high-throughput technology and mapped back to the reference genome.

The comparison of DNaseI hypersensitivity patterns in different datasets can play an important role in the study of gene regulation (Sheffield *et al.*, 2013), for example, in response to a physiological stimulus. However, a major challenge in analyzing these data is the variation in signal-to-noise ratio (SNR) between datasets (Supplementary Table S1). While many potential sources of noise exist, a key variable affecting the SNR is the enzymatic activity of DNaseI, which is difficult to control between experiments. Variation in the level of DNaseI activity leads to different amounts of digestion (Supplementary Fig. S3), altering the composition of the population of short DNA fragments that are sequenced. There is no universal surrogate measurement of digestion that can be used to accurately quantify digestion at the library preparation stage and no ideal control sample (discussed in Supplementary Information, Section S2). For these reasons, distinguishing signal from noise in a manner that allows comparison between datasets is more challenging with DNaseI-seq than with other high-throughput sequencing approaches, such as ChIP-seq.

Several peak-calling programs have been developed for use with high-throughput sequencing data. Most focus on ChIP-seq where a clear input control is available, but some have also been used for DNaseI-seq data, including F-seq (Boyle *et al.*, 2008b), MACS (Zhang *et al.*, 2008) and HOMER (Heinz *et al.*, 2010). While analyzing our own DNaseI-seq data, we found variable performance between peak callers, particularly at low SNRs, and the identification of a suitable peak threshold was challenging. This confounded the comparison between datasets with different SNRs (see Supplementary Information, Section S4 for SNR estimation).

We have developed a peak-calling algorithm (PeaKDEck) that limits the effect of SNR on threshold setting, which is of particular value in datasets with lower SNRs. We have used Hotspot-identified (John *et al.*, 2011) DNaseI-seq sites from 125 cell types published by ENCODE (Thurman *et al.*, 2012) to compare the quality of peak calling by PeaKDEck with that by other peak calling programs. PeaKDEck also includes additional tools for DNaseI-seq data analysis (see Supplementary Information, Section S9 for description of additional tools).

## 2 PEAK CALLING

**Fig. 1.**
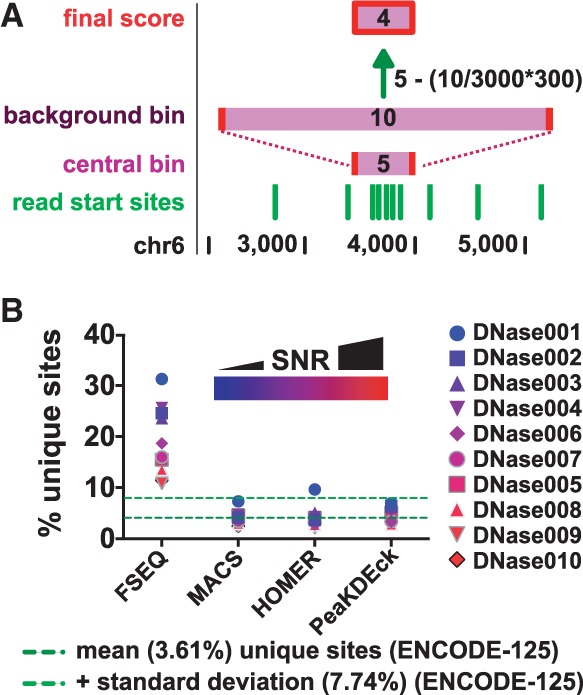
(**A**) PeaKDEck uses a sampling bin to measure signal at any given locus. PeaKDEck calculates the corrected read density by first counting the number of read start sites (green) within a central bin (e.g. Five read start sites in a bin of size 300 bp). Next, the read density in a larger background bin is measured (e.g. 10 reads in a bin of size 3000 bp). Based on this background read density, the expected read density in a bin of central bin size is calculated (e.g. 10 reads in 3000 bp, giving an expected read density of 1 read in 300 bp) and subtracted from the central bin read density to give the corrected read density (4 in this example). (**B**) We calculated the percentage of unique sites identified by four different peak callers in each of 10 sample datasets, and color-coded each dataset based on the SNR from blue (low SNR) to red (high SNR). For datasets with low SNR, PeaKDEck had the lowest percentage of unique peaks out of the four peak callers

The method of peak calling used by PeaKDEck is illustrated in Supplementary Figure S12. First, 50 000 sites are selected randomly from the genome, and overlapping sites are discarded (Supplementary Fig. S12A). Next, the signal strength is measured at the non-overlapping sites using sampling bins (Fig. 1A and Supplementary Fig. S12B). This is achieved by measuring the background read density in a large bin surrounding the site (‘background read density’; default—3000 bp), and then measuring the read density in a smaller focused bin at the same site (‘central read density’; default—300 bp). The corrected read density is calculated by subtracting the expected read density (given the background read density) from the central read density.

Once this calculation has been repeated for all the randomly selected sites in the dataset, a probability distribution is generated to describe the distribution of these corrected read density scores (Supplementary Fig. S12C). Because the distribution of these scores is typically non-Gaussian, PeaKDEck uses kernel density estimation (Supplementary Information, Section S5) to calculate a probability distribution for the randomly selected corrected read density scores, where *n* is the number of sites sampled, *h* is the bandwidth (*h* = 1), *x_i_* is the value of x for the *i*th site and *K_u_* is a Gaussian kernel:

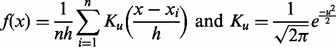



To identify a threshold for peak calling, PeaKDEck calculates the probability that a given corrected read density belongs to the background probability distribution for increasing values of corrected read density. The corrected read density threshold is when the calculated probability drops below a pre-determined level (default—*P* < 0.001). The entire dataset is then scanned in overlapping sampling bins and the corrected read density determined across the genome (Supplementary Fig. S12D). Peaks are called where the corrected read density exceeds the threshold. Peaks can be scored by their maximum corrected read density or probability score.

## 3 PERFORMANCE

We assessed the performance of PeaKDEck by calling peaks in 10 DNaseI-seq datasets from the NCBI Short Reads Archive (Supplementary Information, Section S3). To determine whether the sites identified by PeaKDEck as open chromatin were known open chromatin sites, we amalgamated 125 ENCODE DNaseI-seq datasets for different cell types, tagging each genomic locus with the number of cell types with open chromatin at that site (Supplementary Fig. S5). For each of the 125 datasets, we calculated the percentage of open chromatin sites unique to that dataset, the percentage of sites shared with one other cell type, continuing up to the percentage of sites per dataset shared across all 125 cell types. The mean percentage of unique peaks per dataset was 3.61 ± 4.13% ( ± standard deviation). For the peaks called by PeaKDEck in our 10 sample DNaseI-seq datasets, the mean percentage of unique peaks per dataset was 4.6 ± 1.6% (± standard deviation; see Supplementary Information, Section S6 and S7 for details). This demonstrates that the overlap between open chromatin sites identified by PeaKDEck and known open chromatin sites is within the normal range of variation observed in the ENCODE data.

Because PeaKDEck adjusts signal measurement to account for local variation in read densities and extensively samples background signal in individual datasets, PeaKDEck performs well at setting thresholds in low SNR datasets. To demonstrate this, we called peaks in the 10 sample NCBI DNaseI-seq datasets with PeaKDEck, MACS, FSEQ and HOMER (Supplementary Fig. S13) and quantified the number of unique sites (not occurring in the ENCODE-125 dataset) as a percentage of the total identified sites in each dataset, with each peak caller (Fig. 1B). In the dataset with lowest SNR, 6.95% of the total peaks identified by PeaKDEck were unique, compared with 7.38, 9.64 and 31.4% of peaks identified by MACS, Homer and FSEQ, respectively, suggesting that PeaKDEck is more likely to identify authentic open chromatin sites even at low SNRs compared with other available peak callers.

Although PeaKDEck is designed for use in DNaseI-seq data analysis (using the read start site as the point of interest), it can be used for similar methods such as chromatin immunoprecipitation sequencing and FAIRE-seq, by applying a user-defined offset to calculated genomic positions. PeaKDEck is especially useful compared with other peak callers where SNR is low (see Supplementary Information, Section S8).

*Funding*: Medical Research Council grants (MRC G0901998, MRC G116/165) and National Institute for Health Research Oxford Comprehensive Biomedical Research Centre (BRC) Program.

*Conflict of Interest:* none declared.

## Supplementary Material

Supplementary Data
