# Supplementary material for: PeaKDEck: a kernel density estimator-based peak calling program for DNaseI-seq data
Source: Bioinformatics. 2014 Jan 8;30(9):1302–4. doi: 10.1093/bioinformatics/btt774 (PMC3998130; doi:10.1093/bioinformatics/btt774)
Supplement: Supplementary Data [file supp_30_9_1302__index.html]

PeaKDEck: a kernel density estimator-based peak calling program for DNaseI-seq data — PeaKDEck: a kernel density estimator-based peak calling program for DNaseI-seq data — PeaKDEck: a kernel density estimator-based peak calling program for DNaseI-seq data — Supplementary Data 

# PeaKDEck: a kernel density estimator-based peak calling program for DNaseI-seq data

## Supplementary Data

files

**Files in this Data Supplement:**

- Supplementary Data - pdf file
