# Supplementary material for: PeaKDEck: a kernel density estimator-based peak calling program for DNaseI-seq data
Source: Bioinformatics. 2014 Jan 8;30(9):1302–4. doi: 10.1093/bioinformatics/btt774 (PMC3998130; doi:10.1093/bioinformatics/btt774)
Supplement: Supplementary Data [file supp_btt774_20131217_supplementary_information_unmarked_as_submitted.pdf]

PeaKDEck: a kernel density estimator-based peak calling program for DNaseI-seq data.

Michael T. McCarthy and Christopher A. O'Callaghan

Supplementary Information

Contents

Section S1: The DNaseI-seq method 2
Section S2: Difficulties quantifying DNaseI digestion 3
Section S3: Sample datasets 7
Section S4: Estimation of signal-to-noise ratio in DNaseI-seq data 8
Section S5: Rationale for the use of kernel density estimation 16
Section S6: Peak-calling 18
Section S7: Analysis of dataset-specific unique sites. 22
Section S8: Strengths of PeaKDEck 24
Section S9: Examples of use 25
Section S10: References 26

Section S1: The DNaseI-seq method

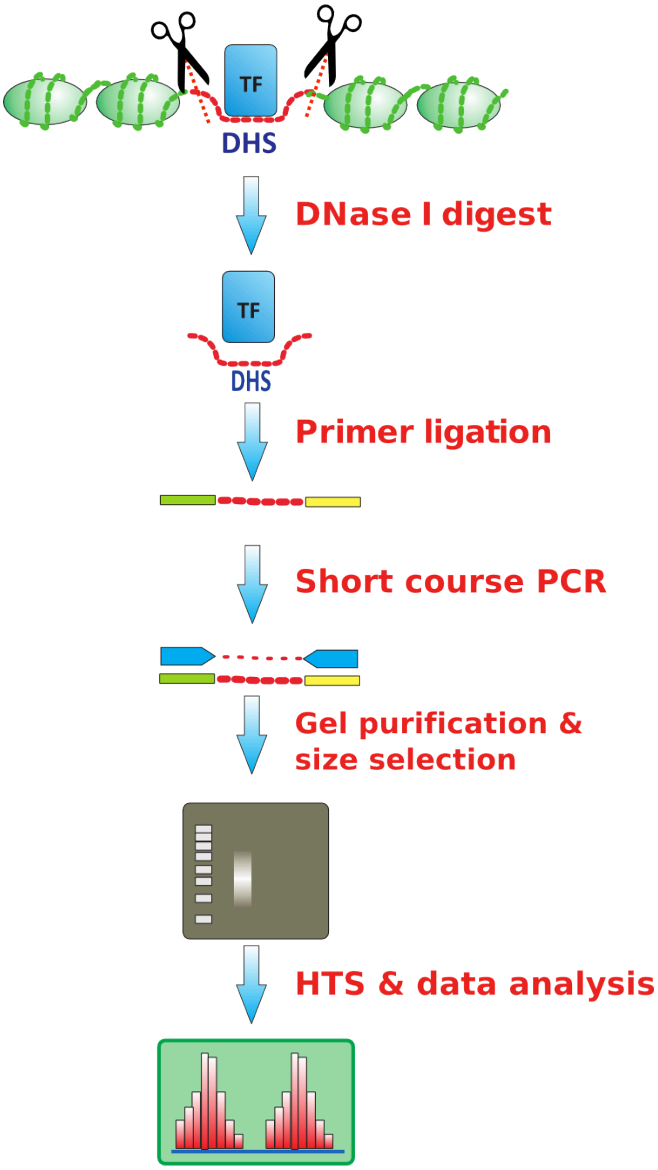

Supplementary Figure S1. The DNaseI-seq method.
In regions of DNaseI-insensitive chromatin, genomic DNA interacts tightly with the histone proteins in the nucleosomes (green ellipses). The histone proteins protect the DNA from digestion by the enzyme DNaseI.
By contrast, in regions of open chromatin, such as those at promoter or enhancer sites, nucleosomes are absent and the genomic DNA is accessible to transcription factors, which bind more loosely than nucleosomes. In open chromatin, the DNA is also accessible to the DNaseI enzyme and so is 'DNaseI hypersensitive'. Where DNaseI cuts twice within a single accessible site, a short fragment of regulatory DNA is released. Adapters are ligated to the released fragments and small fragments are amplified and isolated by gel purification. These fragments are sequenced using high throughput methods.
DHS – DNaseI hypersensitive site; TF – transcription factor;
HTS – high throughput sequencing

Section S2: Difficulties quantifying DNaseI digestion

Although there are several methods for assessing DNaseI digestion (outlined below), there is no universal genome-wide objective measurement that can accurately quantify this digestion, or the signal-to-noise ratio for the digestion before the digested DNA is sequenced. The central problem is that, when differential digestion is detected between samples, it is difficult to distinguish differences in true hypersensitivity from differences in the degree of experimental digestion that is achieved with each sample.

There are two principle methods described for assessing the extent of DNaseI-digestion. Digestion can be qualitatively assessed by pulsed-field gel electrophoresis (Song and Crawford, 2010) (Supplementary Figure S2). While this can be useful, it does not allow accurate quantification of digestion. An alternative approach is DNaseI-Southern blotting which can be combined with qPCR to estimate the degree of digestion at a defined genomic location (McArthur *et al.*, 2001). This approach can be used to estimate DNaseI-hypersensitivity for extended genomic regions (referred to as 'chromatin profiling') (Dorschner *et al.*, 2004; Taylor *et al.*, 2008). Sabo *et al* suggest that optimal digestion for DNaseI hypersensitive site detection is 50-70% copy number loss at hypersensitive sites with respect to insensitive sites (Sabo *et al.*, 2006). We have found this approach helpful for approximate comparison of digestion within experiments (Supplementary Figure S3).

However, a key problem with all these approaches is that DNaseI-hypersensitivity is a dynamic characteristic, and is subject to cell type-specific, experimental and environmental influences (Hernando *et al.*, 2013; Sheffield *et al.*, 2013). Therefore, when differential 'digestion' is detected between samples, it is difficult to distinguish differences in hypersensitivity from differences in digestion. This is particularly relevant when attempting to equalize digestion in different treatment conditions, or between different cell types, and in different experiments, where it is plausible that hypersensitivity itself has changed. The difficulty in quantifying digestion in a standard way is illustrated by the fact that signal-to-noise ratios vary widely between samples in NCBI-deposited DNaseI-seq data (Supplementary Figure S4, Supplementary Table S1; see below for method of signal-to-noise ratio calculation). The use of specific sites as calibration controls is not reliable given these observations.

Phenol-chloroform extracted genomic DNA has been used to generate control data (Sabo *et al.*, 2006; Hesselberth *et al.*, 2009). However, this approach requires the samples to be prepared quite differently, with digestion occurring after DNA extraction, rather than before. While this may have some utility, such a control is unlikely to have an identical background noise pattern to the input sample, and is therefore unlikely to allow for correction of differences in signal-to-noise ratio in the resulting dataset. This is in contrast to similar high throughput methods such as ChIP-seq, where isotype antibodies can be employed in a parallel experiment to obtain highly meaningful control data.

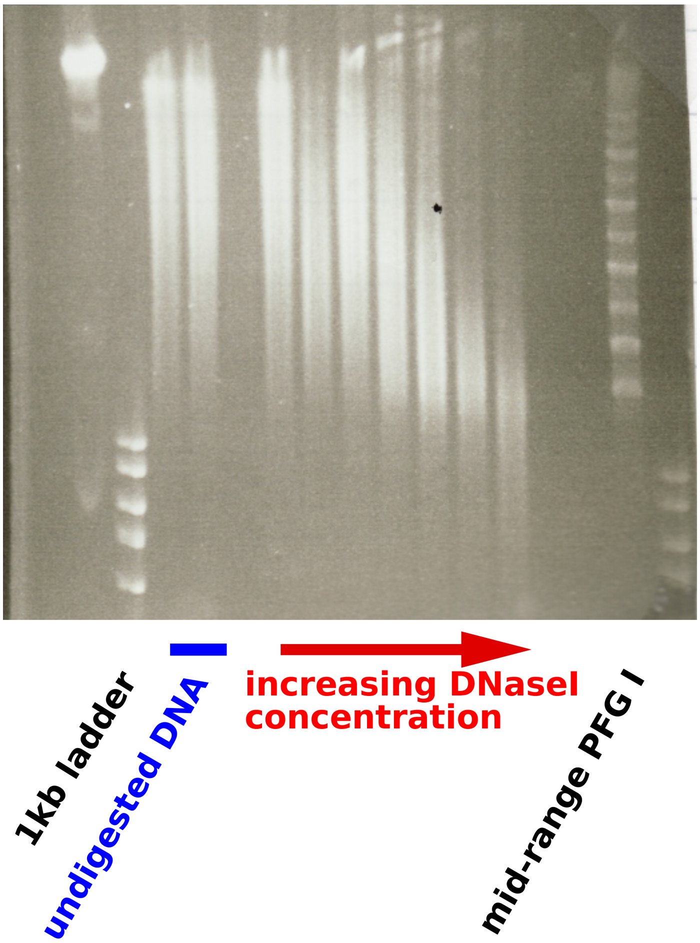

Supplementary Figure S2. Qualitative assessment of digestion by pulsed-field gel electrophoresis.

Digestion of genomic DNA can be assessed qualitatively by pulsed field gel electrophoresis. The above gel shows genomic DNA extracted from MCF7 cells, either undigested (2 lanes) or digested with 1, 2, 4, 8, 16, 32 or 64 units of DNaseI prior to extraction. With increasing digestion, the fragment sizes of the genomic DNA smear decreases gradually. However precise quantification of digestion is not possible.

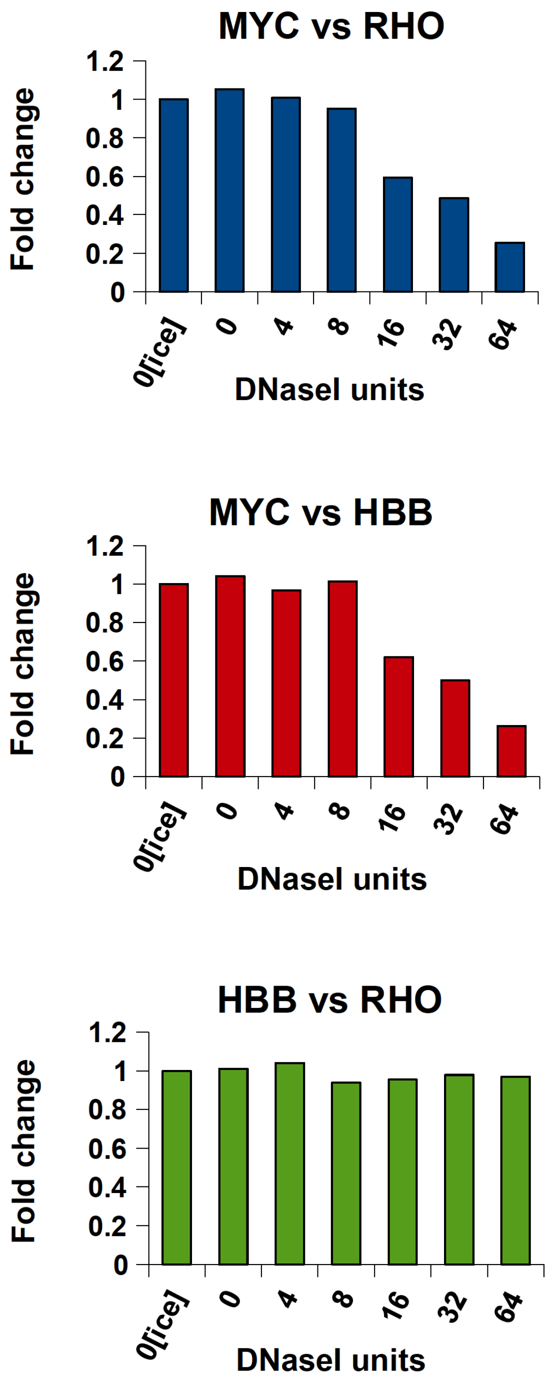

Supplementary Figure S3. DNaseI activity influences signal intensity.

Isolated nuclei from a single population of cells were aliquoted, and digested with different amounts of DNaseI. The DNA was isolated by phenol-chloroform extraction. Primers were made for a known DNaseI hypersensitive site 5' to the c-myc gene (MYC), and for two known DNaseI insensitive sites near the rhodopsin (RHO) and hemoglobin beta (HBB) genes. SYBR green-based qPCR was used to assess relative digestion at the c-myc site compared to digestion at either the insensitive rhodopsin site (blue) or the insensitive beta-globin site (red). This demonstrated increasing digestion of the DNaseI hypersensitive MYC site with higher DNaseI concentrations. Thus signal intensity is influenced by DNaseI activity. No change was observed when we compared the two insensitive sites (green).

Because hypersensitivity at any individual target site (e.g. at the c-myc promoter site) is not a universally stable property, and can change in different cell types, or with different environmental conditions, it is difficult to synchronize digestion levels in different DNaseI-seq libraries prior to sequencing.

All samples were digested at 37 ^{o}C, with the exception of those labeled 0[ice] which were kept on ice throughout (DNaseI is inactive at 0 $^{\circ}$C).

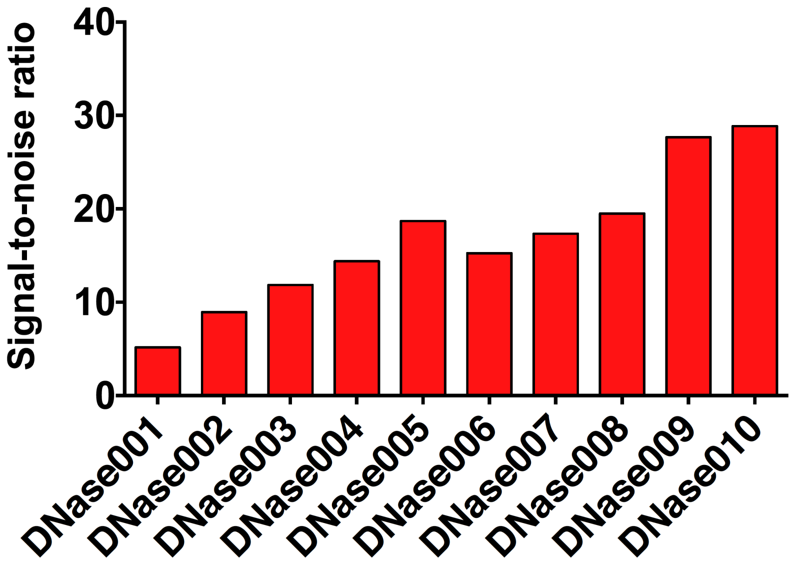

Supplementary Figure S4. Signal-to-noise ratio varies in DNaseI-seq datasets.
To quantify signal-to-noise ratio in 10 DNaseI-seq datasets downloaded from the NCBI Short Reads Archive, we used DNaseI-seq data from ENCODE covering 125 cell types to compile a list of open chromatin elements in the genome, and generate a map of the number of cell types with open chromatin at each site **(Supplementary Figure S5)**. We measured the mean 'signal' intensity, by randomly selecting 50,000 sites that were designated as open chromatin in 60 or more (out of 125) different cell types, and measured the corrected read density at the center of each of these sites. We similarly measured 'noise' by selecting 50,000 loci which were not designated as DNaseI hypersensitive in any ENCODE cell lines. The values depicted are the ratios between mean 'signal' and 'noise' for each of the 10 NCBI sample datasets.

Section S3: Sample datasets

We downloaded datasets from the limited collection of DNaseI-seq datasets available at the time from the NCBI SRA archive (see Supplementary Table S1). Datasets were chosen sequentially, omitting obvious experimental replicates, and, where possible, duplicate cell types to provide sample heterogeneity. Having observed wide variation in signal-to-noise ratio in our preliminary analysis, we wanted to ensure that the test datasets displayed this range of variation. To avoid biases that might arise from choosing biologically identical datasets, and to maintain cell-type heterogeneity, we excluded datasets from duplicate cell lines within the same experimental set. The dataset SRR1013752 is a cell line dataset and was included to widen the heterogeneity of the samples. The full set of datasets, cell types and signal-to-noise ratios are described in Supplementary Table S1. The distribution of signal-to-noise ratio is plotted in Supplementary Figure S9, with included datasets in blue, and excluded datasets in red. For comparison, the ENCODE DNaseI-seq datasets used in our analysis are those described by Thurman *et al* (Thurman *et al.*, 2012).

Label	SRA number	Cell type	SNR	RPB	% of sites shared by >60 ENCODE cell lines
**DNase001**	SRR1013752	MCF7 cell line	5.17	1.03	54.78
**DNase002**	SRR171529	CD56+ primary cells	8.94	1.06	53.91
	SRR171527	CD19+ primary cells	11.27	1.10	45.74
**DNase003**	SRR352544-46	CD4+ primary cells	11.86	1.11	52.92
**DNase004**	SRR171543	CD3+ primary cells	14.38	1.13	53.48
**DNase005**	SRR066352	CD34+ primary cells	18.68	1.18	46.58
**DNase006**	SRR352516-17	CD8+ primary cells	15.25	1.18	60.86
**DNase007**	SRR171576	CD14+ primary cells	17.33	1.20	43.01
	SRR066353	CD34+ cells	20.04	1.21	48.56
**DNase008**	SRR171561	CD3+ primary cells (cord blood)	19.50	1.24	50.91
	SRR066189-90	CD34+ primary cells	21.16	1.29	48.39
	SRR066158-59	CD34+ primary cells	22.33	1.33	50.77
	SRR066175-76	CD34+ primary cells	30.39	1.42	44.20
	SRR203415	CD34+ primary cells	31.32	1.46	43.01
**DNase009**	SRR171574	CD3+ primary cells	27.66	1.52	51.30
**DNase010**	SRR171575	CD8+ primary cells	28.86	1.55	54.28

Supplementary Table S1. Description of all datasets originally assessed.

Section S4: Estimation of signal-to-noise ratio in DNaseI-seq data

While PeaKDEck does not depend on signal-to-noise ratio calculation for peak calling, the estimation of signal-to-noise ratio in DNaseI-seq datasets has some analytical utility. The problem with the calculation of a precise signal-to-noise ratio for DNaseI-seq data is that it would require measurements of signal intensity at sites of true signal (DNaseI hypersensitivity) and at sites of true noise. Unfortunately, no method exists for the precise identification of such sites and so true signal and true noise are not directly measurable.

For this reason, to assess signal-to-noise ratio in a dataset, we instead determine the genomic locations of sites where a body of consistent experimental data indicates that there are high or low probabilities of true signal (DNaseI hypersensitivity) being present. The ratio between the signal at sites of high probability and the sites of low probability is then used to estimate the signal-to-noise ratio.

Estimation of signal. Our approach to the identification of sites with a high probability of true signal is based on analysis of 125 ENCODE DNaseI-seq datasets from 125 different cell lines. We divided the genome into discrete sites, and annotated each site with a count of the number of the ENCODE datasets which had DNaseI hypersensitivity designated within that site (see Supplementary Figure S5). From this annotation, we created 12 lists of genomic sites. The first list contained all sites that were designated as DNaseI hypersensitive in 10 or more cell lines, the next list contained all the sites that were designated as DNaseI hypersensitive in 20 or more cell lines and so on in increments of 10 up to a list of sites that were designated as hypersensitive in 120 or more cell lines. 372,201 sites were designated as hypersensitive in 10 more cell lines, but the number of shared sites fell such that there were only 70,646 hypersensitive sites shared by 60 or more cell lines and only 9039 hypersensitive sites shared by 120 or more cell lines (see Supplementary Table 2).

For each of the 125 individual ENCODE datasets, we then determined the percentage of the sites in each of the 12 groups that were designated as hypersensitive in that individual dataset. So, for example, in dataset #1, 19.5% (72,790 of 372,201) of the sites that were designated as hypersensitive in 10 or more cell lines were hypersensitive in this dataset, 28.8% (63,053 of 218,808) of the sites that were designated as hypersensitive in 20 or more cell lines were hypersensitive in this cell line and so on until 61.4% (5546 of 9039) of the sites that were designated as hypersensitive in 120 or more cell lines were hypersensitive in this cell line (Supplementary Table S2). Having done this for each of the 125 ENCODE datasets we then calculated the mean percentages ± SD across all 125 datasets for the number of sites that were hypersensitive in 10 or more, 20 or more cell lines and so on for each of the 12 lists (Supplementary Figure S6A). The results of this analysis are plotted in Supplementary Figure S6B, where the total number of sites in each of the 12 groups are represented by the full height of the bar (red plus blue) and the mean percentages are shown in red. For example, of the 70,647 sites that are designated as hypersensitive in 60 or more datasets, a mean of 44.0 ± 9.4% of these sites are designated as hypersensitive across the 125 datasets.

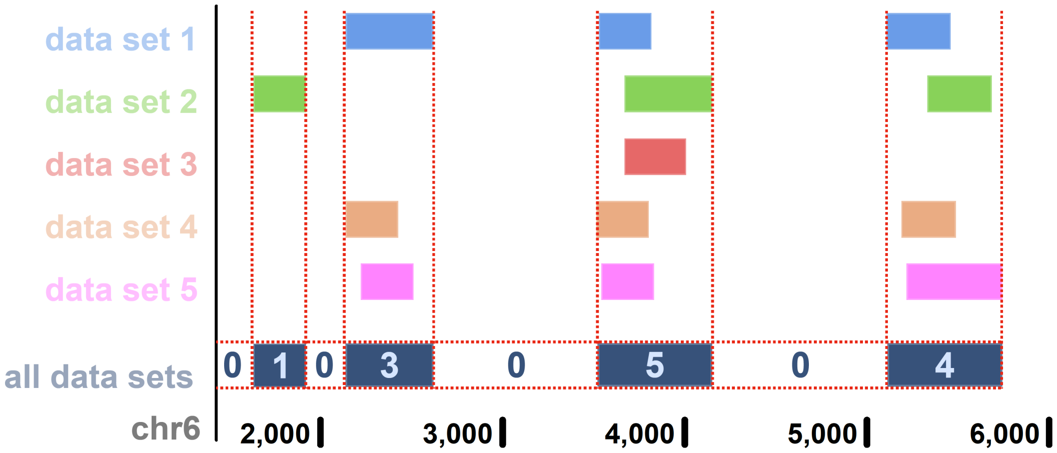

Supplementary Figure S5. Identification of known hypersensitive sites from published ENCODE DNaseI-seq datasets.

To define existing ‘known’ hypersensitive and insensitive sites, we used published ENCODE DNaseI-seq data from 125 cell types (Thurman *et al.*, 2012). The hypersensitive sites from all 125 cell types were amalgamated into one single global track of hypersensitivity. The process of amalgamation is illustrated above. In this example, the hypersensitive sites for 5 cell types at a single locus on chromosome 6 are depicted. Areas of the genome that do not contain any hypersensitive sites are defined, and tagged with a score of zero, indicating no known hypersensitive chromatin at that site. Sites that do contain ENCODE-designated hypersensitivity are assigned a score corresponding to the number of cell types with hypersensitivity in that interval. This data was made into a single track in bed format, as illustrated in the ‘all data sets’ track above.

Group	Total number of sites in group	Number of sites in group with hypersensitivity in ENCODE dataset #1	Percentage of sites in group with hypersensitivity in dataset #1 (%)
**≥10**	372201	72790	19.56
**≥20**	218808	63053	28.82
**≥30**	155763	55669	35.74
**≥40**	117579	49240	41.88
**≥50**	90778	43133	47.52
**≥60**	70647	37062	52.46
**≥70**	55037	30963	56.26
**≥80**	42939	24906	58.00
**≥90**	33559	19649	58.55
**≥100**	26176	15414	58.89
**≥110**	19200	11372	59.23
**≥120**	9039	5546	61.36

Supplementary Table S2. Example of group data calculations.

From this analysis, we chose to estimate 'signal' across the sites that are designated as hypersensitive in 60 or more cell lines. This was because above the 60 level, there was little gain in the percentage of sites that were hypersensitive in each dataset (Supplementary Figure S6A), but the number of sites available for analysis diminished substantially (Supplementary Figure S6B). Ideally, 100% of the sites used to estimate signal in any given dataset would be truly DNaseI hypersensitive. While it is not possible to reach 100% certainty in this respect, in choosing the 70,647 sites that are designated as hypersensitive in 60 or more ENCODE datasets, we know that 44.0 ± 9.4% of these sites are likely to be DNaseI hypersensitive in any given cell type, based on the ENCODE data.

Estimation of noise. To estimate the noise in each dataset, we selected sites with a low probability of containing truly hypersensitive DNA. These 'noise' sites were identified in an analogous manner from the ENCODE data as genomic sites that contain no hypersensitivity signal in any ENCODE cell line.

Calculation of estimated signal-to-noise ratio. The list of 'signal' sites, and the list of 'noise' sites were then used to estimate the signal-to-noise ratio by randomly selecting 50,000 sites from the list of 70,647 signal sites, and 50,000 sites from the list of 'noise' sites. A bin of 300bp was created centered on each of these sites, and any overlapping bins were discarded. The number of reads per base within each bin was calculated for the 'signal' sites, and also for the 'noise, and the mean score across all ~50,000 non-overlapping 'signal' sites was divided by the mean score across all ~50,000 non-overlapping 'noise' sites to arrive at an estimation of the signal-to-noise ratio for that dataset.

If our estimation of signal-to-noise ratio was dominated by the percentage of designated hypersensitive sites in that cell line, then we would expect a linear relationship between the estimated signal-to-noise ratio, and the percentage of the 70,647 sites (designated as hypersensitive in 60 or more ENCODE cell lines) that are designated as hypersensitive in that cell line. This relationship was analyzed for the 10 test datasets. We found no significant

correlation between these values (Figure S6C) which is consistent with our signal-to-noise ratio estimate being driven not by the percentage of designated hypersensitive sites, but by the true signal-to-noise ratio for a dataset.

The choice of 50,000 sites was based on an analysis of our sample datasets, where we found that variability in our estimate of mean signal and noise scores for any given dataset was minimal (coefficient of variation < 1%) when 50,000 or more sites were used for the calculation at both low and high signal-to-noise ratios (Supplementary Figure S7).

To determine whether the estimation of signal-to-noise ratio varies substantially according to which of the original 12 lists of sites were used to estimate 'signal' strength, we repeated the calculation of signal-to-noise ratio as described above for each list of sites. Whether we calculated the signal strength at sites selected from the first list (present in 10 or more cell types) or from the final list (present in 120 or more cell types), the ranking of signal-to-noise ratios from our 10 test dataset changed minimally (Supplementary Figure S8).

Further support for the utility of our approach is obtained by analyzing signal dispersion in the same datasets from the number of reads (or tags) per base with coverage. In high signal-to-noise data sets, the reads will aggregate closely together, and therefore have a higher reads per base score than low signal-to-noise ratio datasets, where reads are more dispersed and spread over a larger number of genomic bases. Plotting these two independent genome-wide measurements for each of our 10 sample datasets (blue), plus 6 additional datasets (red) demonstrated a linear correlation (Supplementary Figure S9), again suggesting that our estimate of signal-to-noise ratio is reasonable.

In summary, precise determination of the signal-to-noise ratio in DNaseI-seq data is not feasible for the reasons outlined above. However, it is possible to estimate signal-to-noise ratio by evaluating signal strength at sites with a high or low probability of being hypersensitive (based on the available experimental data).

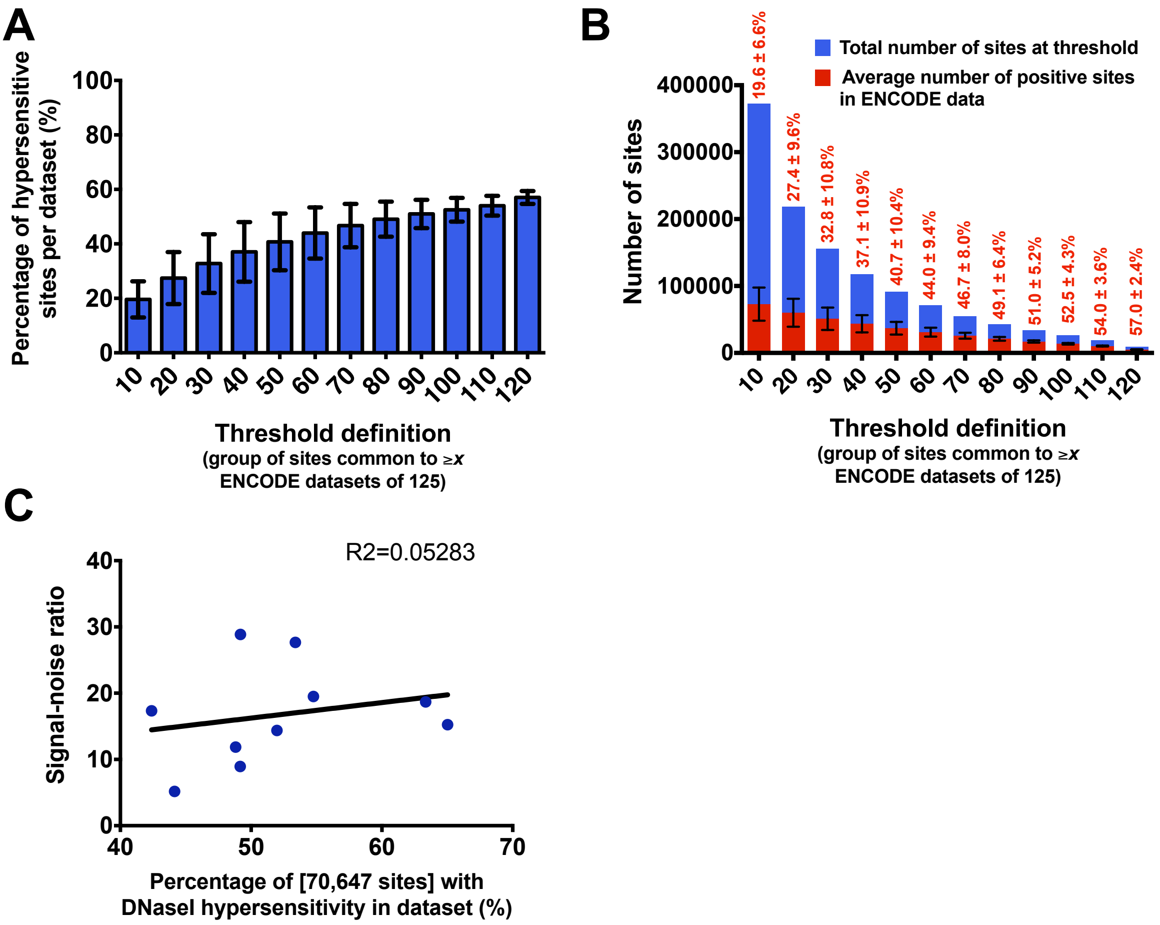

Supplementary Figure S6. Estimation of signal-to-noise ratio.
To identify a suitable list of genomic sites at which to estimate signal for a given dataset for signal-to-noise ratio calculation, we generated 12 lists of genomic sites from the ENCODE-125 dataset, beginning with a list of sites common to 10 or more ENCODE datasets, in increments of 10, up to a list of sites common to 120 or more datasets.

(A) To determine the percentage of sites in each of the 12 lists that were designated as hypersensitive sites, we calculated these percentages for each ENCODE dataset (see Supplementary Information text). Of the 70,647 sites common to 60 or more ENCODE cell types, a mean of 44.0% ± 9.4% of these were hypersensitive across the 125 ENCODE datasets.

(B) The mean number of sites (red bar) ± SD shown in (A) is plotted with the total number of available sites in that category represented by the whole bar (blue plus red). The mean ± SD for each list is itemized in red text.

(C) The estimated signal-to-noise ratio for each sample dataset is plotted against the percentage of the 70,647 sites common to 60 or more ENCODE datasets that contained a designated hypersensitive site in that sample dataset. No significant correlation was detected.

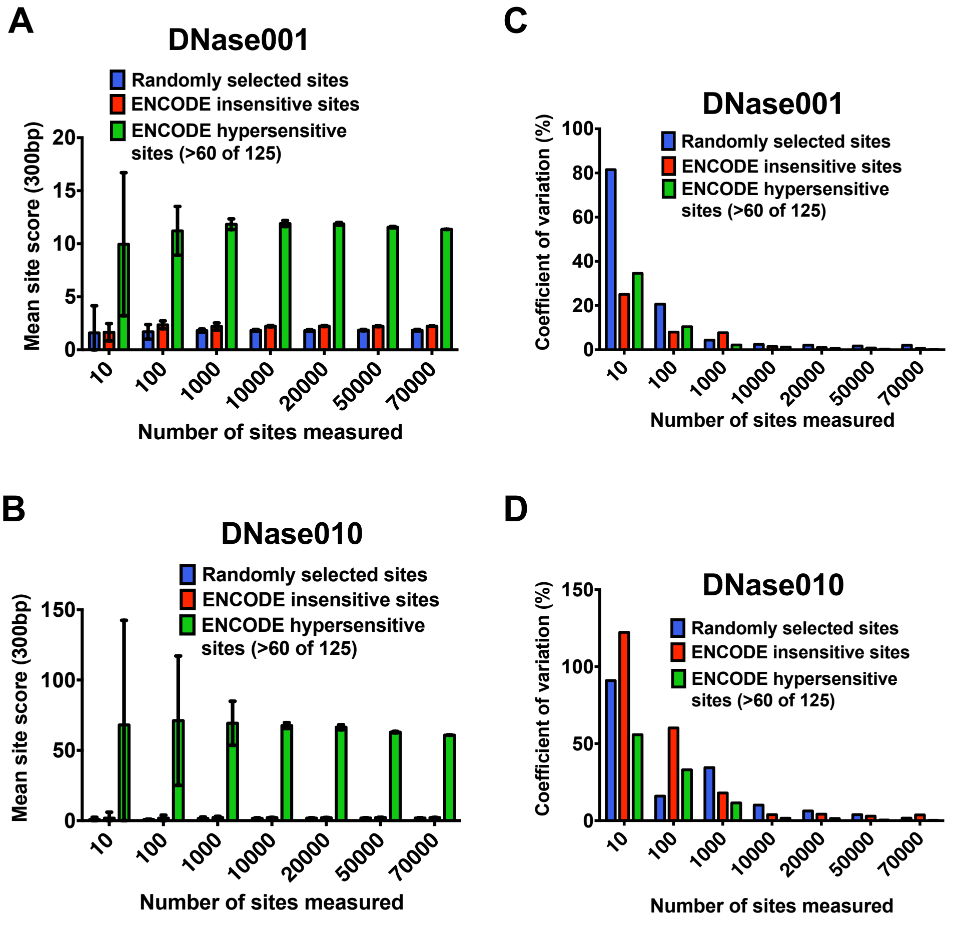

Supplementary Figure S7. Estimates of signal and noise are reproducible in datasets with low and high signal-to-noise ratios.

(A and B) The mean read density scores were calculated for 300bp segments at either randomly selected sites through the genome (blue), sites with no hypersensitivity in the ENCODE dataset (red), or at sites that were hypersensitive in 60 or more ENCODE datasets (green). In each case, the random selection, and measurement of mean read density scores was repeated five times, and the mean ± 95% confidence interval was calculated for both low (A) and high (B) signal-to-noise ratio datasets.

(C and D) When 50,000 or more sites were randomly selected, the coefficient of variation for each of the measured read density scores dropped below 1%, demonstrating that 50,000 sites is a reasonable choice for reproducible SNR estimation.

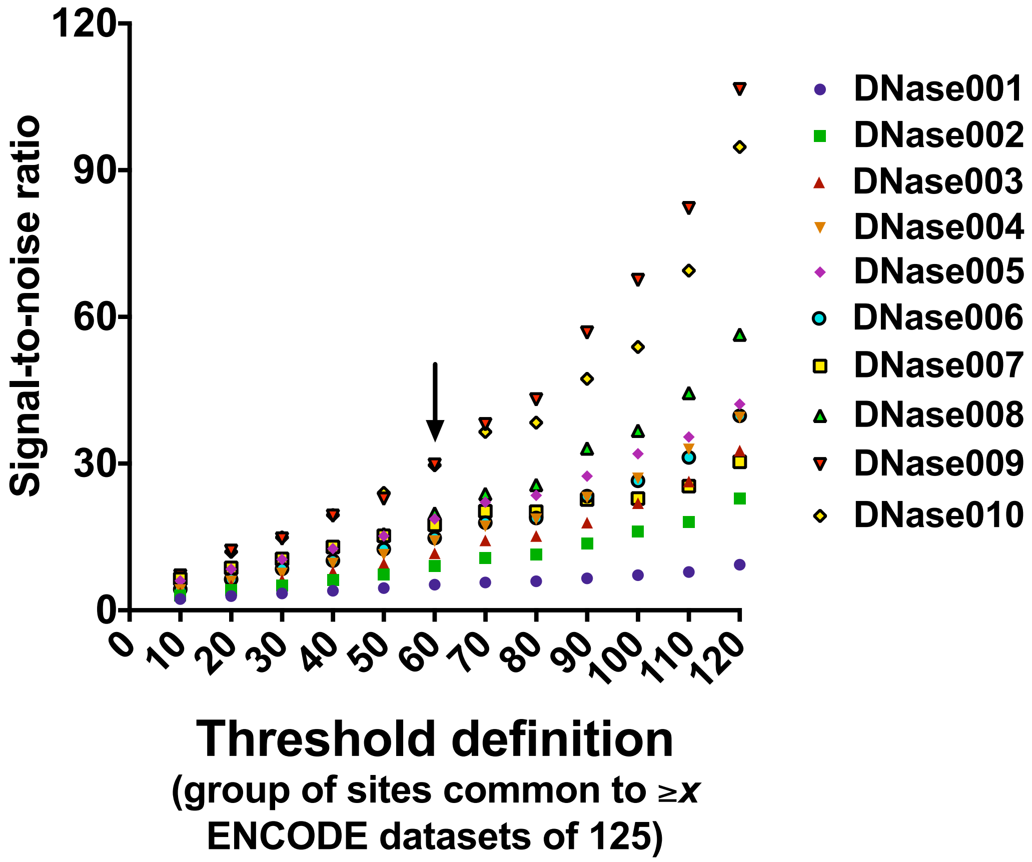

Supplementary Figure S8. Consistency of signal-to-noise ratio estimation.
To test whether measurement of 'signal' at sites common to 60 or more ENCODE datasets had a large impact on our estimation of the signal-to-noise ratio, we plotted signal-to-noise ratio estimates for each of our 10 sample datasets where signal was measured at hypersensitive sites shared by 10 or more ENCODE cell lines, 20 or more cell lines, and so on in increments of 10 up to sites shared by 120 or more ENCODE cell lines. While the magnitude of the signal-to-noise ratio estimate changed, the ranking of signal-to-noise ratio estimates for our 10 sample datasets changed very little, suggesting that the method of estimation of signal-to-noise ratio remains useful, independently of the threshold chosen for defining 'signal' sites

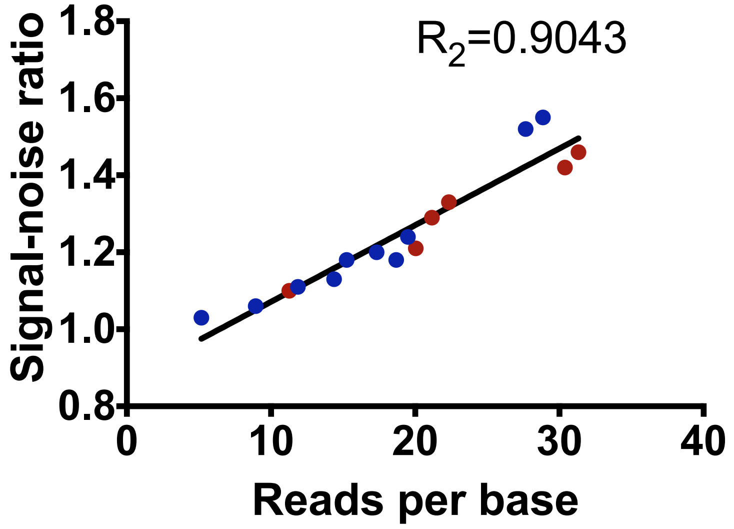

Supplementary Figure S9. The signal-to-noise ratios and reads per base scores in 16 DNaseI-seq datasets.

To test whether our estimate of signal-to-noise ratio was a valid indicator of signal dispersion, we plotted the signal-to-noise ratio estimate for each sample dataset against the number of reads per base covered for the entire dataset. A strong linear correlation was observed, suggesting that our signal-to-noise ratio estimate is a valid measurement of signal dispersion. Datasets included in the analysis in the main text are depicted in blue, and datasets that were excluded due to duplication of cell type or signal-to-noise ratio (to maintain sample heterogeneity) are shown in red.

Section S5: Rationale for the use of kernel density estimation

The critical advantage of using the kernel density estimate (Supplementary Figure S10) over the empirical probability estimate is that it provides a mechanism for smoothing the probability estimate. This smoothed estimate can then be used for rational interpolation of density estimates for points at which data is absent. To demonstrate this, the actual empirical probabilities, and the corresponding kernel estimated probabilities were calculated for low and high signal-to-noise ratio datasets using default PeaKDEck settings (Supplementary Figure S11). A key strength of PeaKDEck is its ability to identify 'safe' thresholds in noisy data, and calling the probabilities in this range is critical. It is clear from Supplementary Figure S11 that threshold estimation would be erratic without smoothing at these probability values. In rare cases, the absence of empirical data for a given read count value may even mean that calculation of an empirical probability would not be possible. The detrimental influence of such (expected) fluctuations in the empirical data is limited by the use of kernel density estimation.

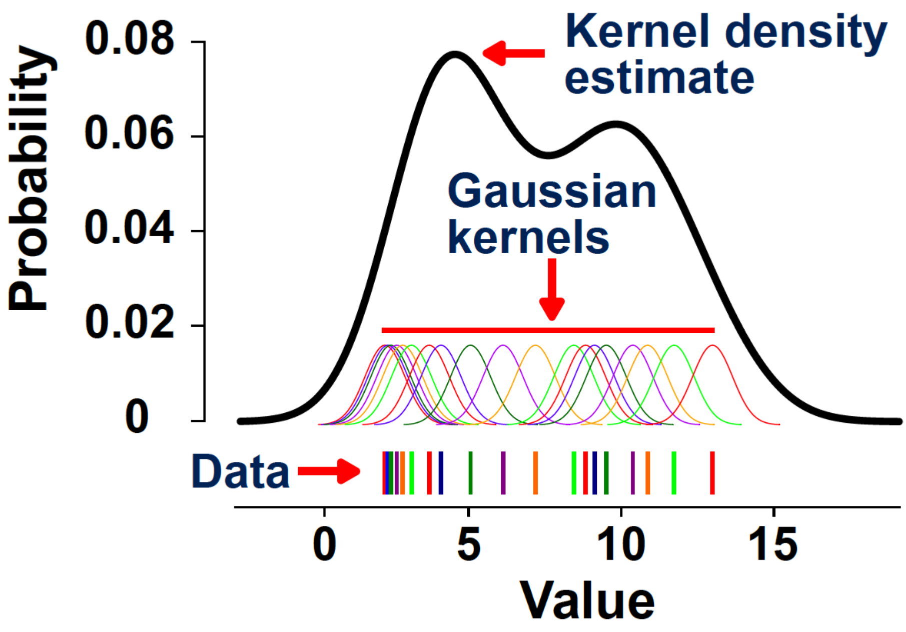

Supplementary Figure S10. Kernel density estimation.
Kernel density estimation is a method for empirically calculating a probability distribution from non-parametric data. PeaKDEck uses Gaussian kernel density estimation to generate a probability distribution based on ~50,000 randomly selected corrected read density measurements from a given dataset. This allows estimation of the probability that any measured corrected read density in the dataset belongs to the random background signal, or is greater than random background signal. By default, PeaKDEck sets the corrected read density threshold for peak calling to a value where the probability that such a density belongs to the random background signal distribution is < 0.001.

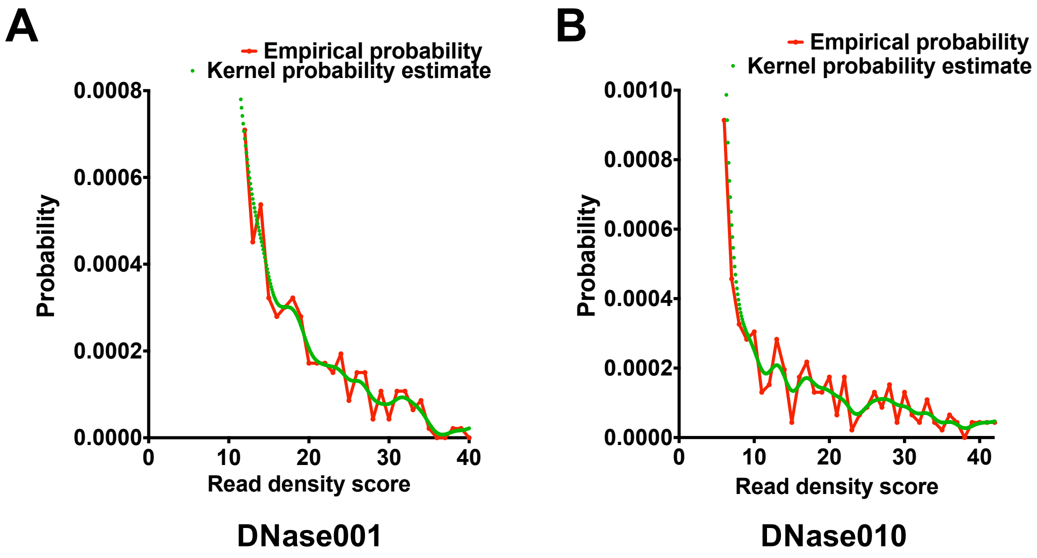

Supplementary Figure S11. Kernel density estimation allows smoothing and interpolation of probability estimates.

To illustrate the distinction between the empirical probability distribution of background read density scores (red) and the calculated kernel density estimated probabilities (green), both these parameters are plotted for a low (A) and high (B) signal-to-noise ratio dataset at the lower end of the read density score probability spectrum. As is evident, a key advantage of the kernel estimated probabilities is smoothing of probability estimates in the lower ranges, and the ability to interpolate results where empirical data is limited.

Section S6: Peak-calling

The approach to peak-calling with PeaKDEck is graphically depicted in Supplementary Figure S12. A quantitative description of PeaKDEck identified peaks is shown in Supplementary Figure S13, and a qualitative description of this data is given in Supplementary Figure S14. Summary results for analysis of our 10 sample datasets are using PeaKDEck are shown in Supplementary Table S3.

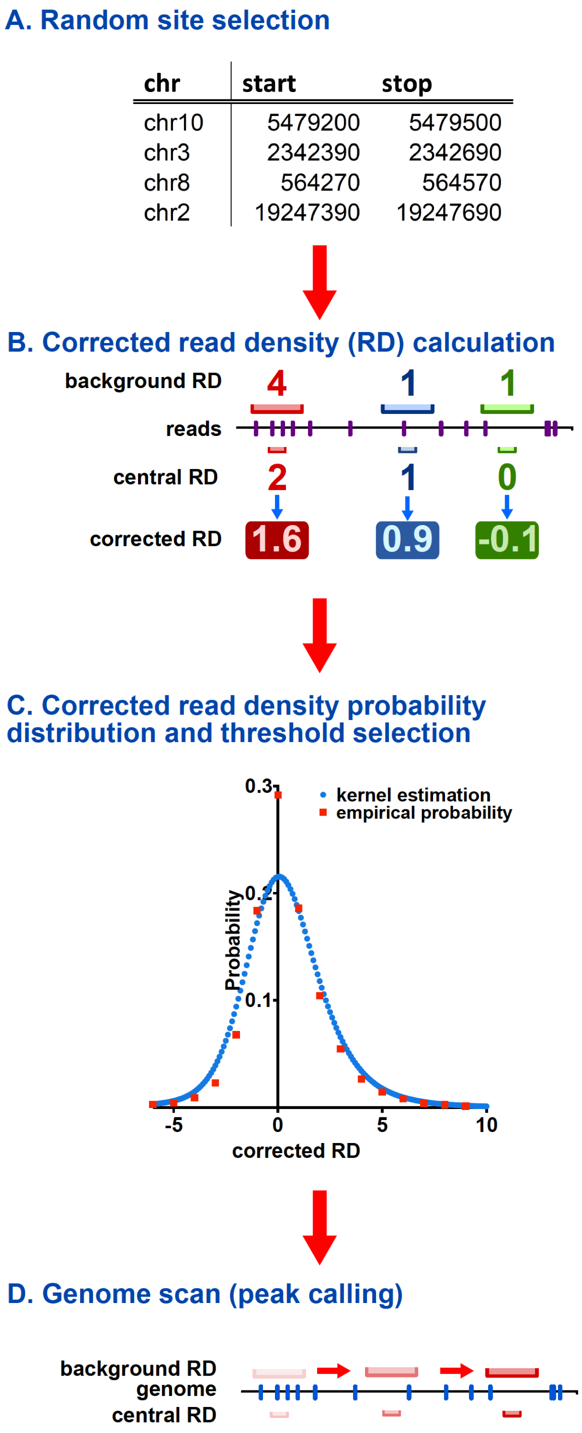

Supplementary Figure S12. PeaKDEck peak calling. The process of peak calling by PeaKDEck can be divided into four steps.

A. First, 50,000 random genomic sites are generated, and overlapping sites are discarded.

B. The read density (RD) at each of these non-overlapping sites (default 300bp) is calculated (central RD). This value is corrected by subtracting the expected background RD calculated from the local background RD (default 3000bp), to give the corrected RD. For the sample calculations illustrated, the background bin is 10 times the size of the central bin.

C. The ~50,000 random corrected RDs are used to calculate a probability distribution of randomly selected corrected RDs, using kernel density estimation (Supplementary Figure 10). The threshold corrected RD for peak calling is calculated from this distribution as the RD for which the probability (P) drops below a cut-off (by default, this value is set at $p < 0.001$).

D. Finally, with this density threshold, the data set is scanned systematically using overlapping bins, and at each position, the corrected RD is calculated as in panel B. A peak is called when this corrected RD is higher than the threshold value.

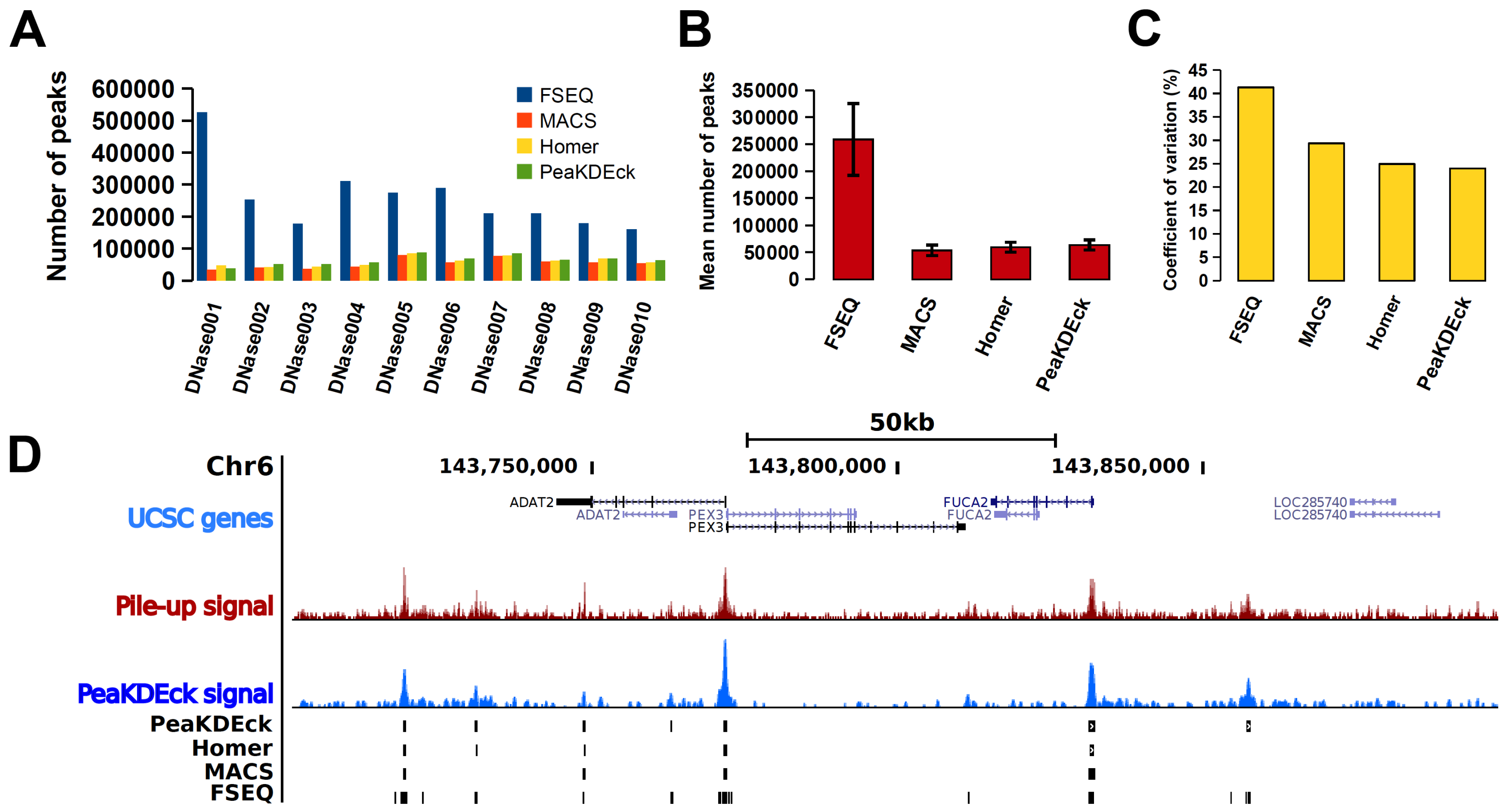

Supplementary Figure S13. Quantitative summary of PeaKDEck comparison. Peaks were called in 10 sample datasets downloaded from the NCBI Short Read Archive, using PeaKDEck, FSEQ, MACS or Homer. (A) The number of peaks identified by PeaKDEck, MACS and Homer was similar for each dataset. The mean number of peaks ± 95% confidence interval for the 10 data sets in the case of each peak caller (B) and the coefficient of variation (C) shows that the number of peaks identified per dataset varies least with PeaKDEck. (D) A sample genome segment, displayed on the UCSC genome browser, shows the Density Analyser output of PeaKDEck (blue), and the peaks called in that segment for each peak caller.

Code	Name	Signal (mean)	Noise (mean)	SNR	Threshold	Peaks	Empirical FDR (%)
**DNase001**	SRR1013752	11.53	2.23	5.17	9.1	37809	0.010
**DNase002**	SRR171529	18.15	2.03	8.94	7.6	51582	0.770
**DNase003**	SRR352544-46	25.61	2.16	11.86	7.7	51625	0.769
**DNase004**	SRR171543	30.63	2.13	14.38	8.0	56213	0.706
**DNase005**	SRR066352	34.94	1.87	18.68	8.9	87055	0.055
**DNase006**	SRR352516-17	30.35	1.99	15.25	7.7	69284	0.573
**DNase007**	SRR171576	33.45	1.93	17.33	7.4	85144	0.466
**DNase008**	SRR171561	39.00	2.00	19.50	7.1	64596	0.615
**DNase009**	SRR171574	60.85	2.20	27.66	6.7	69038	4.239
**DNase010**	SRR171575	63.79	2.21	28.86	6.3	63238	4.629

Supplementary Table S3. PeaKDEck analysis of sample datasets. We downloaded 10 sample data sets from the NCBI Short Read Archive to test PeaKDEck's functions. 'Signal' was calculated by finding the mean background-corrected read density at the center of ~50,000 randomly selected sites that were designated as DNaseI hypersensitive in 60 or more of 125 ENCODE DNaseI-seq datasets. 'Noise' was calculated similarly, using sites identified as not DNaseI hypersensitive in all 125 ENCODE datasets. The empirical false discovery rate was calculated by simulating random DNaseI-seq datasets, and calling peaks at the appropriate threshold for each sample dataset. SNR – Signal-to-noise ratio; FDR – False discovery rate.

Section S7: Analysis of dataset-specific unique sites.

As discussed in the section on 'Estimation of signal-to-noise ratio in DNaseI-seq data', there is no 'gold-standard' approach to the experimental identification (and quantification) of DNaseI hypersensitivity signal on a genome-wide basis, which is independent of DNaseI-seq itself. Because an objectively 'correct' genome-wide quantification of DNaseI hypersensitivity cannot be determined, the validation of peak calling strategies is challenging

We approached the evaluation of peak calling strategy by measuring the percentage of uniquely occurring sites (i.e. sites that do not contain DNaseI hypersensitivity in any of the 125 ENCODE datasets) in each dataset. From our analysis of the ENCODE data, a mean of 0.87 ± 0.73% of the genomic bases in each dataset are designated as hypersensitive. Because the a majority of the genome is not hypersensitive, poorer peak calling strategies are more likely to call peaks randomly in areas of 'noise' as opposed to areas of hypersensitivity signal. Therefore, the percentage of unique peaks in a given dataset is likely to increase as the accuracy of the strategy decreases.

To understand what proportion of sites per dataset is likely to be unique to that dataset, we calculated the mean percentage of unique sites in each of the ENCODE 125 datasets. We found that 3.14 ± 4.13% of ENCODE-designated DNaseI hypersensitive sites per dataset were unique to that dataset. This figure represents a description of the spread of percentages occurring in the large set of ENCODE data which is currently the most standardized and complete data set from which to make this calculation.

When we called peaks in our 10 sample datasets with PeaKDEck, we found that the percentage of unique peaks per dataset varied within the range observed in the larger ENCODE dataset. As discussed above, if our peak calling strategy was poor, we would expect a higher percentage of peaks called by PeaKDEck to be unique to each dataset. Thus our analysis indicates the validity of the PeaKDEck approach.

Threshold calculation and peak calling run times for our 10 sample datasets are shown in Supplementary Table S4.

**Sample**	**Intel core i7-2600; 16GB memory**		**Intel Xeon X5450; 62GB memory**	
	Threshold calc. (s)	Peak calling (s)	Threshold calc. (s)	Peak calling (s)
**DNase001**	67	736	70	946
**DNase002**	63	695	65	901
**DNase003**	65	685	66	902
**DNase004**	62	674	66	909
**DNase005**	62	675	63	908
**DNase006**	61	678	64	918
**DNase007**	58	725	68	971
**DNase008**	64	679	64	945
**DNase009**	63	717	64	918
**DNase010**	61	815	65	963

Supplementary Table S4. PeaKDEck run times on different computing platforms.

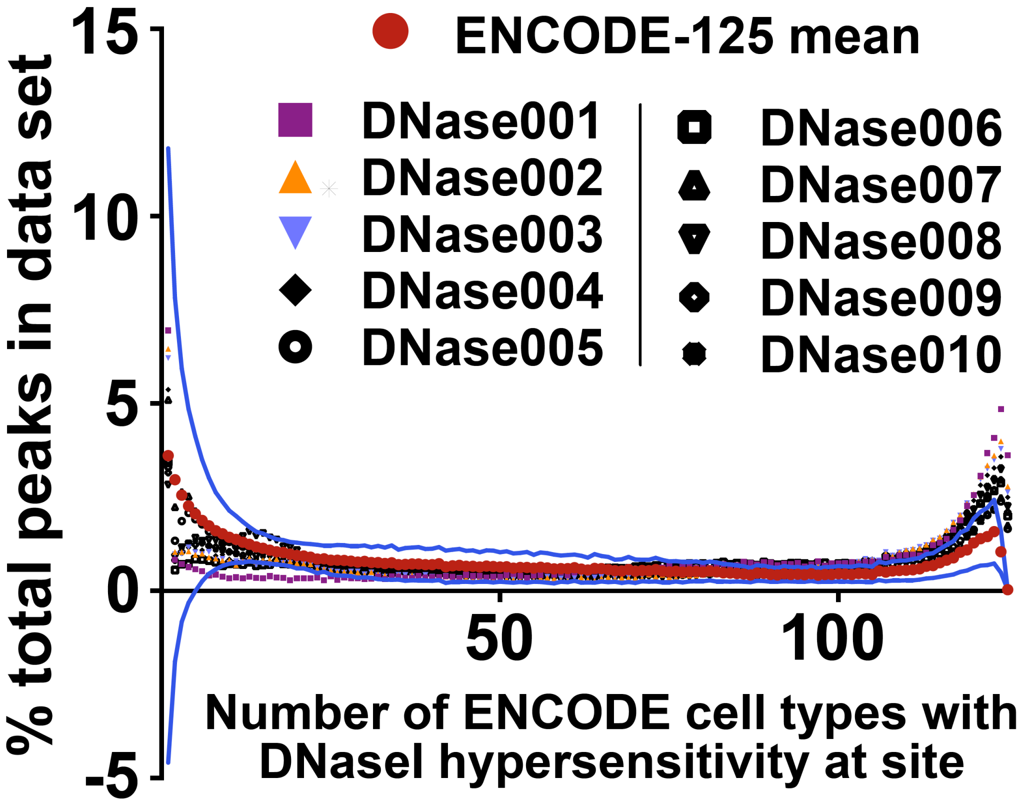

Supplementary Figure S14. Identification of high probability DNaseI hypersensitive sites by PeaKDEck in datasets with different signal-to-noise ratios. The extent to which peaks identified by PeaKDEck in 10 sample data sets overlapped known open chromatin sites was evaluated. For each of the 125 ENCODE datasets, we calculated the percentage of peaks in the dataset that overlapped with peaks in [0, 1, 2, …124] other datasets. The mean percentage of peaks in each category is shown as red dots ± 1.96 standard deviations (blue lines). We then calculated the overlap in peaks called by PeaKDEck for 10 sample DNaseI-seq datasets downloaded from the NCBI Short Reads Archive database **(Supplementary Table S3)**. Importantly, for all datasets, the percentage of unique sites identified by PeaKDEck was within the expected range of variation observed in the 125 ENCODE datasets, even at low signal-noise ratios. This suggests that, sites identified by PeaKDEck are likely to be correctly identified as DNaseI hypersensitive sites.

Section S8: Strengths of PeaKDEck

PeaKDEck has several distinguishing features compared to other peak calling applications. The key features include:

- Use of global background signal frequency distribution for peak threshold calculation
  Extensive sampling of background signal leads to improved ability to distinguish signal from noise at the whole genome and whole dataset level. This facilitates meaningful comparison of different DNaseI-seq datasets.

- Improved peak calling performance at low signal-to-noise ratios
  Kernel density smoothing of sampled background signal allows accurate threshold setting in noisy, low signal-to-noise ratio data. For example, assuming the range of variation in the percentage of unique peaks per dataset in the ENCODE data to be a reasonable estimation of the true variation in the number of unique hypersensitive sites in any given cell type, we calculated the z-scores and probabilities for the percentage of unique peaks in the DNase001 dataset (low signal-to-noise ratio) using each peak caller. For PeaKDEck, 6.95% of peaks were unique, with a 20.9% probability that this value 'belongs' to the normal distribution of values in the ENCODE data set. For MACS, with 7.38% unique peaks, the probability is 18.1%, for HOMER with 9.64%, the probability is 7.2%, and for FSEQ, with 31.40% unique peaks, the probability approaches zero.

- Implementation
  - A full range of tools for analysis of mapped DNaseI-seq data
    Both command line and GUI implementations of PeaKDEck include tools for sorting, filtering and random selection of mapped DNaseI-seq data. In addition, there are tools for generating a density track of the mapped data, and sorting called peaks. To achieve this level of analysis with other peak callers would require several additional software applications.

  - A multi-platform GUI-based implementation
    The GUI application allows usage of PeaKDEck tools without the need for command line access.

  - High accessibility
    The wide variety of command line and GUI multi-platform implementations, combined with its minimal dependencies makes PeaKDEck a widely accessible tool to users of widely varying computational abilities. PeaKDEck does not require any libraries or packages to be installed other than standard Perl for the command line version.

Section S9: Examples of use

The tools available in PeaKDEck are described in Supplementary S5. Examples of use are as follows:

Numerical sorting:
PeaKDEck.pl –NS mappedData.sam > sortedData.sam

Filtering:
PeaKDEck.pl –F sortedData.sam –g hg19.chrom.sizes –q 15 –PCR ON > filteredData.sam

Random read selection:
PeaKDEck.pl –R filteredData.sam –nr 20000000 > filteredData.20m.sam

Density analysis:
PeaKDEck.pl –D filteredData.20m.sam –g hg19.chrom.sizes > densityTrack.wig

Peak calling:
PeaKDEck.pl –P filteredData.20m.sam –g hg19.chrom.sizes > peakList.bed

Size ordering of peaks:
PeaKDEck.pl –T peakList.bed –n 50000 > orderedPeakList.bed

Function	Options	Description
**Sam Filter (-F)**	-q [mapq threshold]<br>-u [uq threshold]<br>-i [include header]<br>-g [chromosome size file]<br>-PCR [remove PCR duplicates (ON|OFF)]	Filters out reads with a mapq score less than (-q) and mismatch score greater than (-uq). The output only includes chromosomes listed in the chromosome size file, in the order in which they are listed. PCR duplicates can be removed by setting –PCR to 'ON'.
**Density Analyzer (-D)**	-t [minimum bin score threshold]<br>-m [maximum bin score threshold]<br>-n [one-sided length of Gaussian function]<br>-s [Gaussian function step size]<br>-d [Gaussian sigma value]<br>-o [track offset]<br>-g [chromosome size file]	Analyzes an ordered mapped reads file in sam format. Assigns a unitless probability density score to each base in the genome, representing its probability of being a hypersensitive site. Only chromosomes given in the chromosome size file are scored.
**Peak Caller (-P)**	-b [blueprint file]<br>-bin [central sampling bin size]<br>-back [background sampling bin size]<br>-npBack [the number of peaks to use for the estimation of background signal]<br>-sig [threshold significance level for peak calling]<br>-STEP [step size]<br>-FLAT [set a flat uncalculated threshold]<br>-g [chromosome size file]<br>-PVAL [peak scoring with p-value (ON|OFF)]	Identifies peaks in mapped target *.sam files. The number of sites sampled to calculate the background signal probability distribution is set using –npBack, and the cutoff threshold for peak calling is calculated at a probability selected by –sig. by default, peaks are scored with the maximum background corrected bin density. These scores can be converted to p-values based on the background signal probability distribution by setting –PVAL to 'ON'.
**Random Read Selector (-R)**	-nr [number of reads]	Randomly selects a target (–nr) number of reads from a given sam file.
**Top Peak Selector (-T)**	-n [number of peaks]	Selects the top (-n) scoring peaks in given bed peak file. Defaults to 'ALL' peaks if –n is not set.
**Numerical Sam Sort (-NS)**		Sorts all reads in given sam file into ascending numerical order by base position.

Supplementary Table S5. List of functions in PeaKDEck.

Section S10: References

Dorschner,M.O. *et al.* (2004) High-throughput localization of functional elements by quantitative chromatin profiling. *Nat Methods*, **1**, 219–225.
Hernando,H. *et al.* (2013) Epstein-Barr virus-mediated transformation of B cells induces global chromatin changes independent to the acquisition of proliferation. *Nucleic Acids Res*, doi:10.1093/nar/gkt886.
Hesselberth,J.R. *et al.* (2009) Global mapping of protein-DNA interactions in vivo by digital genomic footprinting. *Nat Chem Biol*, **6**, 283–289.
McArthur,M. *et al.* (2001) Quantification of DNaseI-sensitivity by real-time PCR: quantitative analysis of DNaseI-hypersensitivity of the mouse beta-globin LCR. *J. Mol. Biol.*, **313**, 27–34.
Sabo,P.J. *et al.* (2006) Genome-scale mapping of DNase I sensitivity in vivo using tiling DNA microarrays. *Nat Methods*, **3**, 511–518.
Sheffield,N.C. *et al.* (2013) Patterns of regulatory activity across diverse human cell types predict tissue identity, transcription factor binding, and long-range interactions. *Genome Res.*, **23**, 777–788.
Song,L. and Crawford,G.E. (2010) DNase-seq: A high-resolution technique for mapping active gene regulatory elements across the genome from mammalian cells. *Cold Spring Harb Protoc*, **5**, 1–11.
Taylor,J.M. *et al.* (2008) Chromatin profiling across the human tumour necrosis factor gene locus reveals a complex, cell type-specific landscape with novel regulatory elements. *Nucleic Acids Res*, **36**, 4845–4862.
Thurman,R.E. *et al.* (2012) The accessible chromatin landscape of the human genome. *Nature*, **489**, 75–82.
